# Drug Induced Liver Injury in Geriatric Patients Detected by a Two-Hospital Prospective Pharmacovigilance Program: A Comprehensive Analysis Using the Roussel Uclaf Causality Assessment Method

**DOI:** 10.3389/fphar.2020.600255

**Published:** 2021-02-05

**Authors:** Laura Pedraza, Olga Laosa, Leocadio Rodríguez-Mañas, Diego F Gutiérrez-Romero, Jesús Frías, José Antonio Carnicero, Elena Ramírez

**Affiliations:** ^1^ Geriatric Research Group, Biomedical Research Foundation at Getafe University Hospital, Getafe, Spain; ^2^ Centre of Network Biomedical Research on Frailty and Healthy Ageing (CIBERFES), Institute of Health Carlos III., Madrid, Spain; ^3^ Division of Geriatrics, University Hospital of Getafe, Getafe, Spain; ^4^ Radiology departments, Virgen del Mar Hospital, Madrid, Spain; ^5^ Clinical Pharmacology departments, La Paz University Hospital, IdiPAZ, School of Medicine, Autonomous University of Madrid, Madrid, Spain; ^6^ Clinical pharmacology department, University Hospital La Paz, La Paz, Spain

**Keywords:** drug-induced liver injury, pharmacovigilance, elderly, adverse drug reaction, roussel uclaf causality assessment method, clinical pharmacology

## Abstract

**Background/aim:** A prospective evaluation of drug-induced liver injury (DILI) in two tertiary hospitals was conducted through a pharmacovigilance program from laboratory signals at hospital (PPLSH) to determine the principal characteristics of DILI in patients older than 65 years, a growing age group worldwide, which is underrepresented in the literature on DILI.

**Methods:** All DILI in patients older than 65 years detected by PPLSH in two hospitals were followed up for 8 years in the La Paz Hospital and 2 years in the Getafe Hospital. A descriptive analysis was conducted that determined the causality of DILI and suspected drugs, the incidence of DILI morbidities, DILI characteristics, laboratory patterns, evolution and outcomes.

**Results:** 458 DILI cases in 441 patients were identified, 31.0% resulting in hospitalisation and 69.0% developing during hospitalisation. The mean age was 76.61 years old (SD, 7.9), and 54.4% were women. The DILI incidence was 76.33/10,000 admissions (95%CI 60.78–95.13). Polypharmacy (taking >4 drugs) was present in 86.84% of patients, 39.68% of whom took >10 drugs. The hepatocellular phenotype was the most frequent type of DILI (53.29%), a higher proportion (65%) had a mild severity index, and, in 55.2% of the evaluated drugs the RUCAM indicated that the causal relationship was highly probable. The most frequently employed drugs were paracetamol (50-cases), amoxicillin-clavulanate (42-cases) and atorvastatin (37-cases). The incidence rate of in-hospital DILI per 10,000 DDDs was highest for piperacillin-tazobactam (66.96/10,000 DDDs). A higher risk of in-hospital DILI was associated with the therapeutic chemical group-J (antiinfectives for systemic use) (OR, 2.65; 95%CI 1.58–4.46) and group-N (central nervous system drugs) (OR, 2.33; 95%CI 1.26–4.31). The patients taking >4 medications presented higher maximum creatinine level (OR, 2.01; 95%CI 1.28–3.15), and the patients taking >10 medications had a higher use of group J drugs (OR, 2.08; 95%IC 1.31–3.32).

**Conclusion:** The incidence rate of DILI in the patients older than 65 years was higher than expected. DILI in elderly patients is mild, has a good outcome, has a hepatocellular pattern, develops during hospitalisation, and prolongs the hospital stay. Knowing the DILI incidence and explanatory factors will help improve the therapy of the elderly population.

## Introduction

Over the last 50 years, drug-induced liver injury (DILI) has been the most frequent reason for withdrawing marketing authorisation for certain drugs (e.g., iproniazid, sitaxentan, and benoxaprofen) (Temple and Himmel, 2002; FDA Drug Induced Liver Injury Rank (DILIrank) Dataset (2020)). DILI has also limited the use of numerous medications (e.g., isoniazid and labetalol) and has generated follow-ups with post-marketing regulatory actions. Several drugs have not been approved in the United States because the European marketing experience revealed the drugs’ hepatotoxicity (Center for Drug Evaluation and Research, 2019). Most of the drugs recalled due to hepatotoxicity have caused death or resulted in the need of transplantation at rates of ≤1 per 10,000 (Larrey, 2000). Therefore, the typical drug development databases with thousands of patients exposed to a new drug will show no cases. Only the most overt hepatotoxins can be expected to show cases of severe DILI in the 1,000–3,000 patients typically studied and reported in a new drug application, and drugs that cause such predictable and dose-related injury are generally discovered and rejected during preclinical testing. More difficult to detect is the toxicity that is unpredictable or not dose-related that occurs at doses that are well tolerated by most people but seems to depend on individual susceptibilities that have not as yet been characterised (Council for International Organizations of Medical Sciences, 2019).

As age increases, the risk of liver injury also increases (Larrey, 2002; Lucena et al., 2020; Danjuma et al., 2020; Chalasani and Bjornsson, 2010). A Japanese study evaluated the clinical characteristics of elderly patients, and concluded that, in addition to the association with advanced age, there was a high number of prescribed medications, uncertain medication duration, and longer exposure time to certain drugs. In terms of outcomes, the patients had longer hospitalisations, a greater need of intensive treatment and less accuracy in the diagnosis (Onji et al., 2009). However, Meier et al. (Meier et al., 2005) concluded from a prospective study that there was no significant relationship between comorbidity/polypharmacy and the risk of DILI. Other reviews (Herrlinger and Klotz, 2001; Cotreau et al., 2005), indicated that advanced age might affect the clearance of certain cytochrome P450 substrates, altering the activity or expression of phase I or phase II drug-metabolising enzymes.

Since 2007, the La Paz University Hospital has employed the pharmacovigilance program from laboratory signals at hospital (PPLSH), which uses automatic laboratory signals (ALS) as Tegeder et al. (Tegeder et al., 1999) described, to monitor a large number of patients with limited resources. This support tool for detecting adverse drug reactions (ADR) in hospital has proven useful for detecting and evaluating serious adverse drug reactions (SADR) associated with increased morbidity and lengthened hospital stays, and for gathering the necessary detailed information to study the risk factors associated with these SADRs (Ramirez et al., 2010; Ramirez et al., 2013; Ramirez et al., 2017; Ramirez et al., 2019). With the collaboration of La Paz University Hospital this programme was implemented in the Getafe University Hospital, starting in the geriatric ward.

Due to the increasingly older adult population worldwide, there has been a growing rate of polypharmacy, DILI and ADRs. Older adults tend to use significantly more concomitant drugs, which is likely due to the increased number of underlying diseases and conflicting information regarding the diseases. The objective of this study was to detect all DILI in patients older than 65 years through a proactive and prospective pharmacovigilance program in two Spanish hospitals during the patients’ follow-up periods to describe the factors related to DILI.

## Materials and Methods

### Setting

At the time of the study, electronic clinical records (ECRs) included all laboratory data, imaging, and other exploratory results, previous medical reports and discharge summaries. A specific database application was developed within the Integrated Laboratory System (Labtrack), which has been available in the La Paz University Hospital since 2006, to detect predefined abnormal laboratory signals (ALS). The program employed in the Getafe University hospital is a system that integrates the results of the hospital’s central laboratory (ServoLab Laboratory Computer System, Version 3), after requesting the required permits. All ALS were retrieved systematically. The approval for publishing the programme was obtained from the Institutional Review Boards at La Paz University Hospital and at Getafe University Hospital.

### Definition of Automatic Laboratory Signal

Table 1 Lists of abnormal laboratory signals (ALS) criteria (EMEA, 2010).

**TABLE 1:** Definition of automatic laboratory signals employed to detect drug-induced liver injury.

Alanine aminotransferase (ALAT) × 3 times the upper limit of normal (ULN) or
Alkaline phosphatase (ALP) level × 2 ULN or
Gamma glutamyl transpeptidase (GGT) × 2 ULN or Bilirubin × 2 times the ULN
Total bilirubin (TB) levels × 2 times the ULN

### Observation Periods

The observation periods by hospital were as follows:

Getafe University Hospital: 31/Oct/2016 to 01/Jan/2018 in the geriatric ward.
La Paz University Hospital: 01/Jul/2007 to 31/Dec/2015 in the entire hospital.

The prospective follow-up of the cases was conducted for at least 1 year.

### Detection, Evaluation and Notification

The procedure for detecting and evaluating ADRs has been described elsewhere. Ramirez et al. (2010) Briefly, in **phase I**, on-file laboratory data at admission or during hospitalisation were screened 7 days a week, 24 h a day. In **phase II**, the patients were identified to avoid duplicates, and the ECRs were reviewed. In **phase III**, a case-by-case evaluation was performed for the remaining cases. For the cases in which an ALS was detected during the hospital admissions of patients over 65 years of age, the ALS was analysed using the ECRs. When a clear alternative cause was ascertained, the case was considered non-drug related. For the remaining cases, two physicians from the Clinical Pharmacology Department conducted a detailed review of patients’ records, a patient visit and/or interview with their relatives to obtain more detail and, if necessary, further tests. When a SADR was suspected, a withdrawal of the suspected drugs was discussed with the attending physician, and the patient was followed-up during hospitalisation and referred to a pharmacovigilance consultation. For all patients categorised as having a DILI, a complete adverse reaction report was submitted to the pharmacovigilance centre in Madrid.

### Drug-Induced Liver Injury Definition

DILI identification was made in accordance to the criteria defined by the CDER-PhRMA-AASLD Conference, 2000 (EMEA, 2010) and the severity definition employed in the hospital, was the one described by the Harmonized tripartite guidelines of the International Conference on Harmonisation of Technical Requirements for Registration of Pharmaceuticals for Human Use (ICH, 2003).

### Causality Assessment

The Roussel Uclaf Causality Assessment Method (RUCAM 1993) (Danan and Benichou, 1993), the most commonly employed diagnostic algorithm for assessing causality in DILI (Danan and Teschke, 2018; Teschke, 2018), includes weighted scoring of an event according to 7 distinct domains related to the temporal relationship between exposure to a particular drug and the liver injury (both its onset and course), the exclusion of alternative non-drug-related aetiologies, exposure to other medications that could explain the DILI, risk factors of the adverse hepatic reaction, evidence in the literature regarding DILI from the drug in question and response to re-exposure to the medication. The total score (ranging from −7 to +14) from the domain-specific assessment classifies the event as highly probable (>8), probable (6–8), possible (3–5), unlikely (1–2) or excluded (<0), based on the likelihood of a DILI (21). The categories of highly probable, probable, and possible were considered drug related.

### Collection of Patient Data

All notifications and comorbidities (hypertension, diabetes mellitus, dyslipidaemia, toxic habits), basic demographic data (sex, age, weight, height, use of concomitant medications), the timing of the signal (during hospitalisation or causing hospitalisation), the number of drugs consumed at the time of the signal and the characteristics of the DILI and hospital stays, presence of chronification (abnormal laboratory results sustained for more than 3 months), type/pattern of DILI, RUCAM classification, severity (mild, moderate, severe or fatal) and whether the DILI and SADR were recorded in the patients’ discharge reports were recorded. Also, the suspected drugs (at the start and end of treatment) were recorded according to the Anatomical Therapeutic Chemical (ATC) classification system and outcome. Polypharmacy was defined as the use of more than four drugs in the DILI onset.

### Drug Consumption

Drug consumption was characterised at defined daily doses (DDD), which is the standard adult dose of a drug for 1 d treatment, as defined by the World Health Organisation’s ATC classification system. DDDs were calculated for cases of DILI that occurred during hospitalisation.

### Laboratory Test Results

The laboratory variables were recorded at three time points (baseline, maximum or peak, and outcome recovery) and included: Alanine aminotransferase (ALT), alkaline phosphatase (ALP), total bilirubin (TB), gamma-glutamyl transferase (GGT), thromboplastin activity, lactate dehydrogenase, creatinine, albumin, blood pH and eosinophils. The increase above the limit of normal for all laboratory variables was calculated.

### Data Analysis

In-hospital incidence rate of DILI was calculated by dividing the number of cases of drug-induced reactions by the total number of hospitalised patients older than 65 years during the prospective follow-up. The uncertainty of association was assessed by calculating the 95% two-sided Poisson confidence interval.

Patients’ mean of stay was compared with the mean department stay (i.e. the mean stay for all patients in that department). The result was a positive or negative number depending on whether the patients spent more or fewer days in hospital than expected per department per year, which allowed us to report the length of the hospitalisation according to whether the DILI occurred during hospitalisation or caused the hospitalisation.

Incidence rate for DILI per 10,000 DDDs was calculated by multiplying the number of DDDs by the mean number of days each drug was consumed. The result was divided by the consumption of each drug (in DDDs) in the hospital during the study period.

Descriptive data were presented as means (Standard Deviation) and N (%, proportions). The quantitative variables are presented as mean and standard deviation (SD), and the qualitative variables are presented as absolute and relative frequencies. Then, a bivariate analysis was performed to determine the variables potentially related to the onset of DILI. For the related qualitative variables, Chi-squared test was employed; and for quantitative variables, Student’s t-test or and the Mann-Whitney U test, were employed as appropriate.

A receiver operating characteristic (ROC) curve was constructed to evaluate the explanatory factors associated with type, severity, DILI timing (in-hospital or before) and polypharmacy in the DILI cases. To obtain the most parsimonious model, a Backward stepwise procedure was performed, which started with a model using all confounding variables available in the database and removing one after the other until all the remaining variables included in the model were significant. This procedure was used in logistic regression model using as dependent variable the following variables recoded as follows:

DILI type (hepatocellular vs. cholestatic/mixed)

‐ Severity (severe / moderate vs. mild).
‐ DILI (in-hospital vs. resulting in hospitalisation).
‐ Polypharmacy.

Cross-validation of the final models of the logistic regression was made by dividing them into 4, 5, and 10 groups (fold cross-validation), to assess the reproducibility of the statistical differences, and the model’s variance estimation. The data analyses were performed using IBM SPSS Statistics version 20.0.0 (IBM Corporation, United States).

## Results

### Incidence and Length of Stay

**FIGURE 1:**
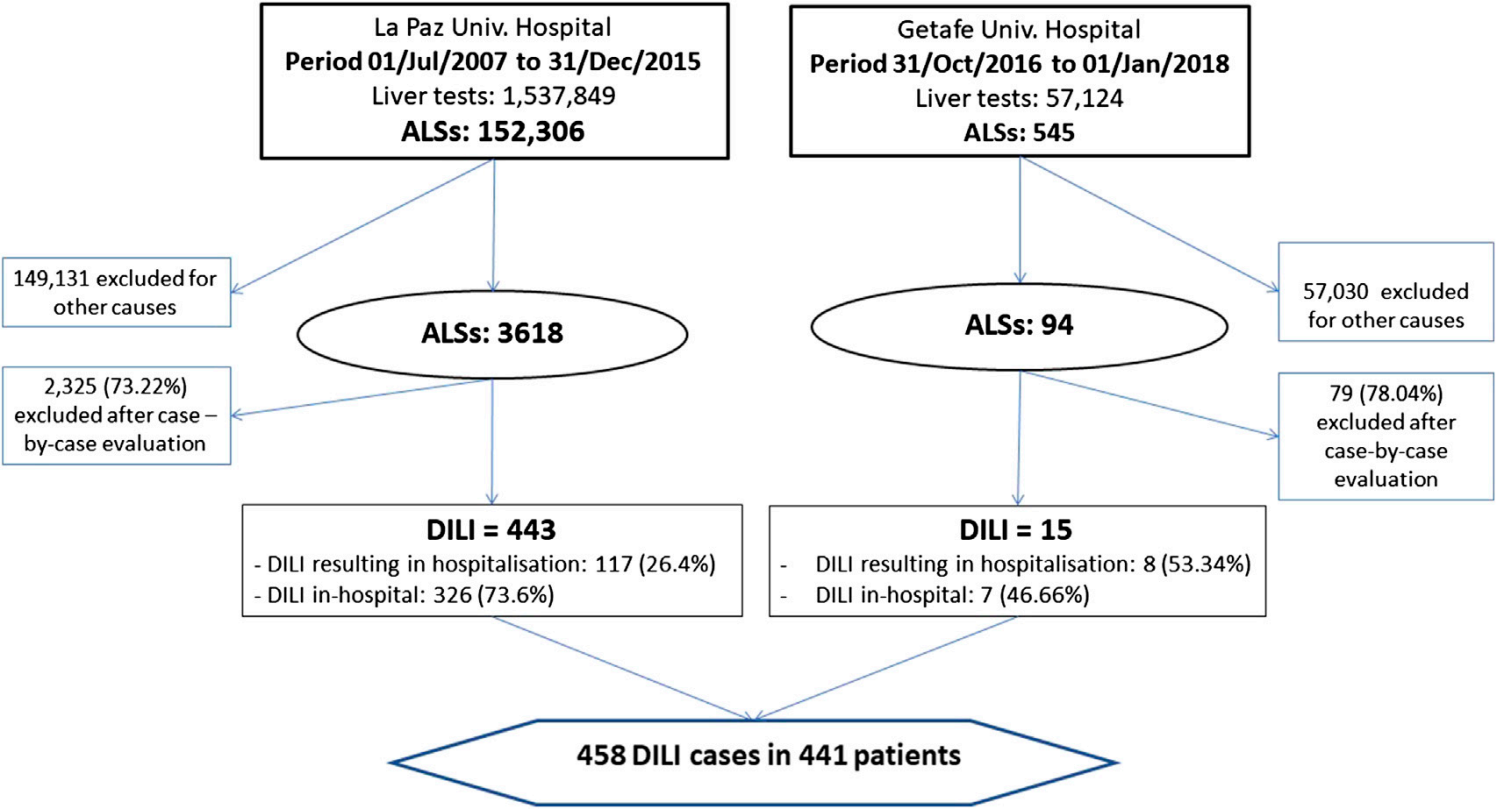
Flowchart of drug-induced liver injury cases detection. Abbreviations: ALSs, automatic laboratory signals.

During the study period, 1,594,973 liver tests were processed (GPT/ALT, GGT, ALP or TB), of these, 3,712 met ALS criteria, and a total of 458 cases in 441 patients were categorised as DILI. Figure 1 shows the flowchart e of the PPLSH during the study period. In the Getafe University Hospital, 15 (15.96%) cases of DILI were detected in 15 patients (8 cases resulting in hospitalisation and 7 in-hospital), obtaining a positive predictive value of 15.95%. In the geriatrics department, there were 2041 hospitalisations during the study period. The incidence of DILI was 73.49/10,000 hospitalisations (95% CI 58.11–91.79 per 10,000). In the La Paz Hospital, 443 (12.2%) cases of DILI were detected in 413 patients (117 cases resulting in hospitalisation and 326 in-hospital), obtaining a positive predictive value of 12.24%. Of these, 17 patients had more than one DILI episode (11 patients had 2 episodes, 1 patient had 3 episodes, and 1 patient had 5 episodes). Of these, 16 cases occurred in the geriatrics department. The incidence of DILI in the geriatrics department was 79.17/10,000 (95% CI 63.44–98.46 per 10,000). There was no significant difference between the cases of DILI detected in the two hospitals. The overall incidence rate of DILI was 76.33/10,000 admissions (95% CI 60.78–95.13 per 10,000).

**FIGURE 2:**
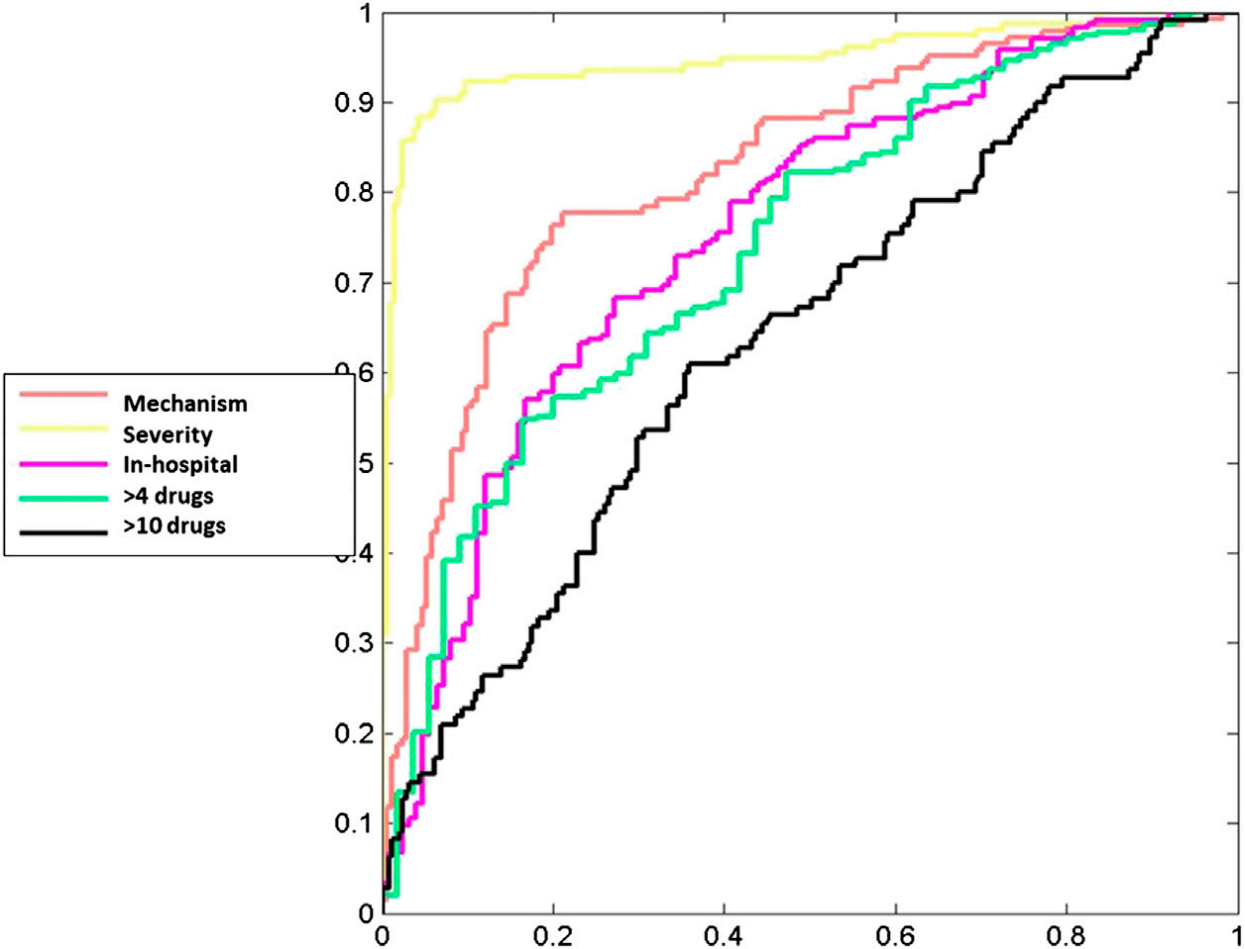
ROC curves for the dependent variables.

The most frequent DILI (69.0%) cases were those arising during hospitalisation. The mean length of stay of the patients admitted with a DILI was 14.4 days (+6.44 more days than the mean hospital length of stay), and the mean excess stay due to DILI was + 17.9 days. Table 2 shows the mean excess stay due to DILI (DILI resulting in hospitalisation or in-hospital) and by department. Compared with in-hospital DILI cases, greater severity (moderate or severe or fatal) (24.7% vs. 54.9%, *p* < 0.001), and worse outcome (sequel or death) (11.27% vs. 1.6%, *p* = 0.015), were observed in DILI cases resulting in hospitalisation.

**TABLE 2:** Prolongation of hospitalisation by medical department.

	DILI RH (*n*)		DILI IH (*n*)	
	dd	(+/−d)	dd	(+/−d)
All medical departments (*n* = 458)	142		316	
	8.41	(+6.44)	8.52	(+17.9)
Internal medicine (*n* = 120)	45		75	
	9.29	(+4.60)	9.27	(+17.06)
Traumatology (*n* = 67)	2		65	
	3.6	(+27.4)	3.42	(+21.2)
Others (*n* = 53)	25		28	
	6.66	(+6.26)	7.61	(+25.75)
Haematology (*n* = 39)	5		34	
	14.28	(−2.88)	15.6	(+13.24)
Pneumology (*n* = 36)	2		34	
	11.05	(+6.45)	10.8	(+10.3)
Gastroenterology (*n* = 29)	27		2	
	7.1	(+12.5)	7.3	(+0.7)
Geriatrics (*n* = 27)	12		15	
	3.79	(+0.79)	5.15	(+8.36)
Cardiology (*n* = 26)	5		21	
	6.54	(−0.54)	6.43	(+11.66)
Oncology (*n* = 25)	15		10	
	11.3	(+0.17)	10.9	(+7.2)
Neurology (*n* = 21)	1		20	
	9.1	(+1.9)	8.89	(+32.21)
Surgery (*n* = 15)	4		11	
	6.45	(+36.3)	7.36	(+21.45)

DILI, drug-induced liver injury; DILI RH, mean DILI stays resulting in hospitalisation; DILI IH, mean DILI in-hospital stays; dd, mean department stay during the study period; +/−d, prolongation of hospitalisation in days.

### Characteristics of Patients With Drug-Induced Liver Injury

The mean age of the patients with DILI was 76.85 (SD 7.9) years, and 240 were women (54.4%). Of the 441 patients, 9.52% had a history of ADR and 8.4% had previous liver disease. Pre-existing liver disease was associated to a greater severity (fatal 5%, severe 10%, moderate 35%, and mild 50%; *p* = 0.042). Table 3 lists the characteristics of the patients with DILI. Polypharmacy was present in 86.84% of DILI patients, of which 39.68% received more than 10 drugs. Table 4 lists the characteristic of the DILI cases. The hepatocellular phenotype was the most frequent type of DILI (53.29%), a higher proportion (65%) of cases were mild (increased ALT levels without jaundice), and the overall DILI-related mortality was 3.27%. Table 5 lists the mean number of times above the upper limit for the laboratory parameters of the DILI cases. Table 6 lists the characteristics of DILI cases by sex and age. The age was dichotomized to ≤ 75 vs. > 75 years old. The frequency of DILI in patients older than 75 years was higher than in younger patients (54.37% vs. 45.63%, respectively). There were more women (58.63% vs. 48.33%, respectively) with DILI, and a higher proportion of polypharmacy in those older than 75 years (88.35% vs. 77.99%).

**TABLE 3:** General characteristics of the patients with drug-induced liver injury.

Variable			
Number of patients, *n*		441	
Age, years, mean (SD)		76.85	(7.92)
Female sex, *n* (%)		240	(54.42)
Hospital stay, days, mean (SD)		14.49	(24.15)
Number of drugs, mean (SD)		8.67	(4.21)
Polypharmacy *, *n* (%)		383	(86.84)
Patients taking 5–10 drugs		208	(47.16)
10 drugs		175	(39.68)
History of ADR, *n* (%)	No	399	(90.48)
	Yes	42	(9.52)
Previous liver disease	No	403	(91.59)
	Yes	37	(8.41)
Weight (kg), mean (SD)		70.49	(15.12)
Height (cm), mean (SD)		162.93	(75.02)
Blood albumin, mean (SD)		3.25	(0.55)
BMI, mean (SD)		27.72	(5.48)
Hypertension, *n* (%)	No	125	(28.34)
	Yes	316	(71.66)
Dyslipemia, *n* (%)	No	262	(59.41)
	Yes	179	(40.59)
Diabetes, *n* (%)	No	314	(71.20)
	Yes	127	(28.80)
Smoking habit, *n* (%)	No	301	(68.25)
	Current	42	(9.52)
	Former	98	(22.22)
Alcoholic habit, *n* (%)	No	369	(83.67)
	Current	52	(11.79)
	Former	20	(4.54)

Polypharmacy * >4 concomitant drugs.

ADR, adverse drug reaction; BMI, body mass index; SD, standard deviation.

**TABLE 4:** General characteristics of drug-induced liver injury cases.

Variable			
Number of cases		458	
DILI during hospitalisation		316	(69)
DILI type	Hepatocellular	243	(53.29)
	Mixed	86	(18.86)
	Cholestatic	127	(27.85)
RUCAM classification	Highly probable	253	(55.24)
	Probable	204	(44.54)
	Possible	1	(0.22)
Severity	Mild	298	(65.06)
	Moderate	109	(23.79)
	Severe	36	(7.86)
	Fatal	15	(3.27)
Outcome	Recovered	399	(87.11)
	Transplantation	0	(0.00)
	Death	15	(3.27)
	Unrelated death	41	(8.95)
	Sequelae	3	(0.66)
Hepatitis chronification	No chronification	451	(98.47)
	Chronification	7	(1.53)
Recorded as hypertransaminasemia in DR	No	192	(42.11)
	Yes	264	(57.89)
DILI recorded in DR	No	292	(64.04)
	Yes	164	(35.96)

Values are number of cases, *n* (%).

ADR, adverse drug reaction; DR, discharge records; DILI, drug induced liver injury; RUCAM, Roussel Uclaf Causality Assessment Method.

**TABLE 5:** Means number of times above upper limit of normality for the laboratory parameters.

Laboratory parameter		Number of times above the ULN	
ALT, U/L (NR, 3.40–4.9)	Baseline	0.6	(0.4)
	Maximum	11.0	(18.5)
	Recovered	1.7	(7.5)
Change per day in ALT	Baseline–Maximum	0.92	(3.28)
	Baseline–Recovered	0.08	(0.74)
	Maximum–Recovered	−0.93	(5.40)
LDH, U/L (NR, 12–78)	Baseline	1.3	(0.9)
	Maximum	5.7	(16.9)
	Recovered	1	(3.8)
Change per day in LDH	Baseline–Maximum	0.49	(3.09)
	Baseline–Recovered	0.02	(0.41)
	Maximum–Recovered	−0.62	(2.85)
ALP, U/L (NR, 84–246)	Baseline	2.2	(23.5)
	Maximum	4.0	(5.7)
	Recovered	1.5	(1.4)
Change per day in ALP	Baseline–Maximum	0.13	(0.26)
	Baseline–Recovered	0.01	(0.08)
	Maximum–Recovered	−0.10	(0.25)
Creatinine, mg/dLNR, 0.5–1.20)	Baseline	1.1	(0.4)
	Maximum	3.2	(21.3)
	Recovered	1.1	(0.6)
Change per day in creatinine level	Baseline–Maximum	0.38	(5.48)
	Baseline–Recovered	0.002	(0.03)
	Maximum–Recovered	−0.26	(2.05)
Total bilirubin, mg/dl (NR, 0–1)	Baseline	1.4	(2.1)
	Maximum	6.2	(11.4)
	Recovered	1.9	(5.0)
Change per day in total bilirubin level	Baseline–Maximum	0.48	(1.35)
	Baseline–Recovered	0.01	(0.11)
	Maximum–Recovered	−0.44	(0.67)
GGT, U/L (NR, 5–55)	Baseline	1.9	(2.7)
	Maximum	17.5	(21.5)
	Recovered	4.2	(7.3)
Change per day in GGT level	Baseline–Maximum	0.87	(2.04)
	Baseline–Recovered	0.06	(0.23)
	Maximum–Recovered	−0.62	(1.32)
TPAC (%)(NR, 70–110)	Baseline	1.1	(0.2)
	Maximum	0.7	(0.3)
	Recovered	1.0	(0.2)
Change per day in TPAC	Baseline–Maximum	−0.05	(0.08)
	Baseline–Recovered	−0.001	(0.02)
	Maximum–Recovered	0.06	(0.12)
Blood pH (7.35–7.45)	Baseline	1.0	(0.01)
	Maximum	1.0	(0.02)
	Recovered	4.0	(53.6)
Change per day in pH	Baseline–Maximum	−0.001	(0.01)
	Baseline–Recovered	0.0001	(0.002)
	Maximum–Recovered	0.004	(0.01)
Eosinophil count, 10³/µl (NR, 0–0.5)	Baseline	0.4	(0.5)
	Maximum	2.0	(5.1)
	Recovered	0.5	(1.3)
Change per day in eosinophil level	Baseline–Maximum	0.22	(1.04)
	Baseline–Recovered	0.01	(0.12)
	Maximum - Recovered	−0.22	(0.78)

*Values are listed as mean ± SD, unless otherwise noted.

ADR, adverse drug reaction; ALP, alkaline phosphatase; DILI, drug-induced liver injury; ALT, alanine aminotransferase; GGT, gamma-glutamyl transferase; LDH, lactate dehydrogenase; NR, normal range; SD, standard deviation; TPAC, thromboplastin activity; ULN, upper limit of normality.

**TABLE 6:** Characteristics of DILI cases by sex and age.

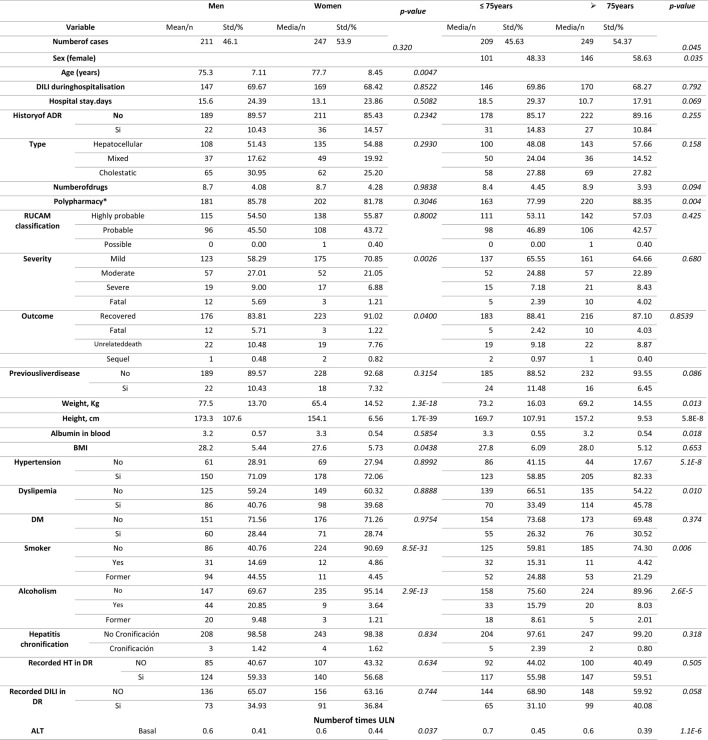

### Culprit Drugs

There were 582 culprit drugs. Of the 458 cases, 166 (36.24%) involved two or more suspicious drugs. The most frequently used drugs were paracetamol (50 cases), followed by amoxicillin-clavulanate (42 cases), atorvastatin (37 cases), cephazolin and levofloxacin (both 21 cases), metamizole (18 cases), and meropenem (17 cases). Table 7 lists the characteristic of the DILI cases per drug, for the most common culprit drugs. In 55.2% of the evaluated drugs the RUCAM indicated that the causal relationship was highly probable. A statistically significant increase in ALT was observed with levofloxacin, and a significant total bilirubin maximum was observed with amoxicillin-clavulanate (Table 7). The incidence rate of in-hospital DILI per 10,000 DDDs was highest for piperacillin-tazobactam (66.96/10,000 DDDs), followed by meropenem (56.6/10,000DDDs), and atorvastatin (37.05/10,000DDDs) (Table 8). Table 9 lists the characteristics of the DILI cases per ATC group. Group J of anti-infective drugs for systemic use (34.5%) followed by group N of drugs for the central nervous system (20.8%) and group C of the cardiovascular system (13.4%) were the therapeutic groups most frequently associated with DILI cases. Groups J and L (antineoplastic agents and immunomodulators) were associated with a higher percentage of polypharmacy. Group A (digestive system and metabolism) was associated with greater severity (severe and fatal). Outcome recovery creatinine level was significantly higher in group C.

**TABLE 7:** Characteristic of drug-induced liver injury per drug for the most common culprit drugs.

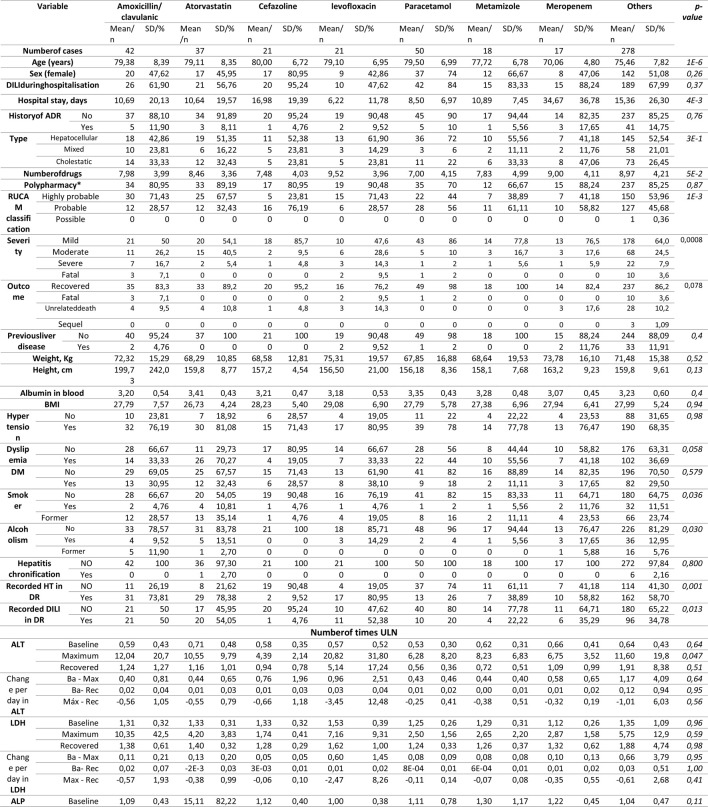

**TABLE 8:** Incidence by consumption in cases of in-hospital drug-induced liver injury.

Drugs	No. of HULP/DH Cases	ATC	DDD (U) VIA	Consumption in DDDs in DILI DH *		Consumption during the study period (DDDs)	Incidence rate (per 10,000 DDDs)	95% CI (per 10,000 DDDs)
Piperacillin-Tazobactam	15	J01CR05	14 (g) P	138.83	310,987.11		66.96	51.92–83.97
Meropenem	15	J01DH02	3 (g) P	30.38	80,506.56		56.60	43.17–72.72
Atorvastatin	21	C10AA05	20 (mg) O	38.05	215,664.00		37.05	1.6–11.66
Ceftriaxone	15	J01DD04	2 (g) P	13.5	58,930.00		34.36	25.21–49.84
Cephazolin	20	J01DB04	3 (g) P	8.4	55,506.00		30.27	17.79–39.28
Amoxicillin Clavulanic	27	J01CR02 P1	1.5 (g) 0 3 (g) P	10.92	127,664.02		23.09	4.11–17.08
Methotrexate	10	L04AX03	2.5 (mg) O	6.7	32,435.43		20.66	51.04–83.97
Paracetamol	42	N02BE01	3 (g) O/P/R	27.72	843,477.48		13.80	7.65–22.23
Enoxaparin	15	B01AB05	2 (U) P	58.33	815,045.61		10.73	5.49–18.39
Dexketoprofen	16	M01AE17	75 (mg) O/P	8.45	242,935.29		5.57	2.20–11.67
Levofloxacin	10	J01MA12	0.5 (g) P/O	2.31	70,420.00		3.28	1.09–8.77
Metamizole	15	N02BB02	3 (g) P/O/R	12	2,342,993.88		0.77	0.02–3.69

DILI only during hospitalization; All consumption calculations are in grams.

DILI, drug-induced liver injury; DH, during hospitalization; ATC, anatomical, therapeutic, chemical classification system; DDD, defined daily dose; CI, confidence interval; mg, milligrams; g, grams; O, oral; P, parenteral; R, rectal; U, international unit.

**TABLE 9:** Characteristic of drug-induced liver injury per ATC group.

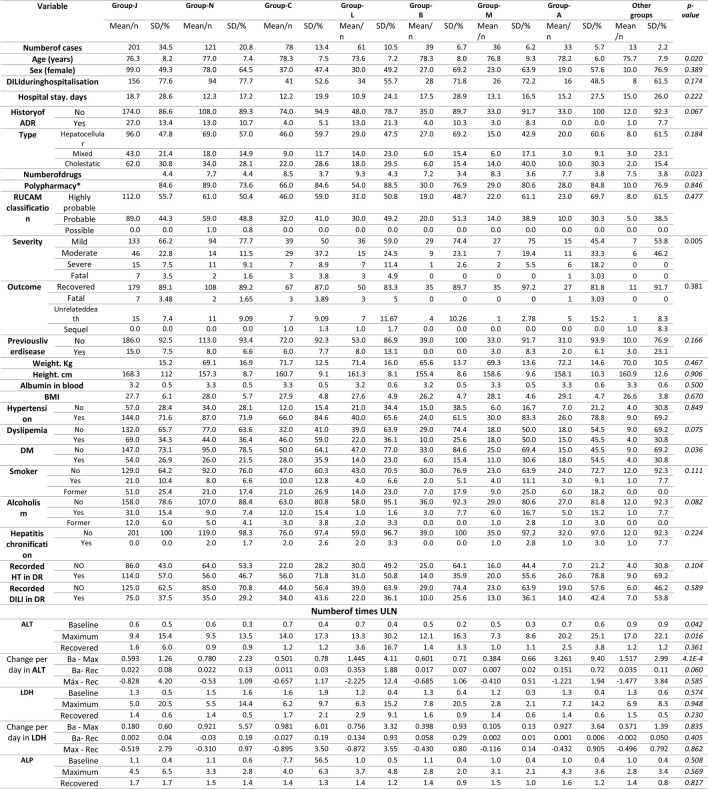

### Relevant Factors of Logistic Regression Models

Table 10 shows the final variables included of the logistic regression models. Several regression models were constructed: A) According to the pattern, the discriminant factors for the hepatocellular pattern diagnostic (vs. cholestatic/mixed) were maximum ALT level, maximum ALP level and baseline GGT level with OR (95% CI) per increase of an unit were 1.069 (1.04–1.10), 0.789 (0.702–0.887) and 0.673 (0.541–0.837), respectively; B) according to severity, an increment in one unit in maximum ALT level and an increment of one unit in the maximum TB had a higher risk of severe DILI of 1.05 (CI 1.2–1.08) and 4.14(2.98–5.76), respectively; C) the use of drugs included in the ATC group J of antiinfectives for systemic use and group N of drugs for the central nervous system had a higher risk of in-hospital DILI. On the other hand, an increment of one unit in maximum ALT level and an increment in one unit in maximum TB level had a lower risk of in-hospital DILI (vs. causing hospitalisation). The OR (95% CI) were 2.65 (1.58–4.46), 2.33 (1.26–4.31), 0.963 (0.945–0.982) and 0.941 (0.911–0.973), respectively; D) in DILI due to polypharmacy maximum creatinine levels, baseline TB level and the use of group N were associated with taking more than four drugs. Our results showed that maximum creatinine level was a risk factor with an OR (95% CI) for an increment of one unit of 2.01 (1-0.28-3.15), whilst in the case of baseline TB level and taking group N drugs were protective factors with OR (95% CI) of 0.744 (0.554–1.00) and 0.488 (0.240–0.835) per increment in one unit in TB levels and the use of group N, respectively; and E) DILI in patients taking more than 10 drugs, the use of group J drugs, , baseline creatinine levels and maximum BT level were associated with the event Our results showed that taking group J drugs and baseline creatinine level were risk factor with OR (95%CI) of2.08 (1.31–3.32), 1.78 (1.02–3.1) for the use of group J drugs and per increment in one unit in baseline level, respectively. On the other hand, maximum TB level was a protective factor with an OR (95% CI) of 0.946 (0.904–0.990) for an increment of one unit in this parameter. Figure 2 shows the ROC curves of the parsimonious logistic regression models for the considered outcomes (polypharmacy, severity, in-hospital and pattern of DILI).

**TABLE 10:** Saturated models of logistic regression (explanatory variables, odds ratio and 95% confidence interval, area under the curve) of: (A) Type of DILI; (B) DILI severity; (C) DILI occurring during hospitalization vs. resulting in hospitalization; (D) DILI by > 4 drugs vs. ≤ 4 drugs; and (E) DILI by > 10 drugs vs. ≤ 10 drugs.

Variable	*p*-value	OR	LL	UL
(A) Type (hepatocellular vs. Cholestatic/mixed)				
Max ALT (U/L)	1.9E−05	1.069	1.037	1.102
Max ALP (U/L)	7.5E−05	0.789	0.702	0.887
Baseline GGT (U/L)	3.7E−04	0.673	0.541	0.837
AUC		0.825	0.777	0.872
(B) Severity (severe vs. moderate/mild)				
Max ALT(U/L)	2.14E−04	1.050	1.023	1.078
Max TB (mg/dl)	2.55E−17	4.146	2.983	5.763
AUC		0.949	0.924	0.974
(C) DILI during vs. resulting in hospitalization				
Group J	2.3E−04	2.652	1.578	4.459
Group N	0.007	2.331	1.259	4.314
Max ALT (U/L)	1.4E−04	0.963	0.945	0.982
Max TB (U/L)	3.0E−04	0.941	0.911	0.973
AUC		0.756	0.711	0.800
(D) DILI by > 4 drugs vs. ≤ 4 drugs				
Group N	0.012	0.448	0.240	0.835
Max creatinine (mg/dl)	0.002	2.007	1.279	3.149
Baseline TB (mg/dl)	0.050	0.744	0.554	1.000
AUC		0.739	0.685	0.793
(E) DILI by > 10 drugs vs. ≤ 10 drugs				
Group J	0.002	2.083	1.307	3.320
Baseline creatinine (mg/dl)	0.044	1.779	1.015	3.119
Max BT (mg/dl)	0.017	0.946	0.904	0.990
AUC		0.638	0.582	0.694

AUC, area under the curve; ALT, aminotransferase; ALP, alkaline phosphatase; GGT, gamma-glutamyl transferase; Group J, anti-infectives for systemic use; Group N, central nervous system drugs; LL, lower limit of 95% confidence interval; OR, odds ratio; TB, total bilirubin; *p*-value, statistical significance; UL, upper limiter of 95% confidence interval.

For hepatocellular DILI, the model including maximum ALT, maximum ALP and GGT levels had a model performance AUC (95%CI) of 0.820 (0.777–0.872). For severe DILI, the model including maximum ALT and maximum TB levels had a model performance of 0.949 (0.924–0.974). For in-hospital DILI, the model including use of Group J drugs, use of Group N drugs, maximum ALT and maximum TB levels had a performance of 0.756 (0.711–0.800). For DILI related by more than four drugs, the model including use of Group N drugs, maximum creatinine and baseline TB levels had a performance of 0.739 (0.685–0.793). And finally, for DILI related to more than 10 drugs, the model including the use of group J drugs, baseline creatinine and maximum TB levels had a performance of 0.638 (0.582–0.694). Table 11 shows the performance (AUC (95%CI)) for the parsimonious logistic regression models using all data and cross-validation algorithms. Non-significant cross-validation results indicated a high reproducibility of the models.

**TABLE 11:** Performance for the parsimonious logistic models using all data and cross-validation algorithms.

Variable	Validation of final models								
	Model			Validation 4 fold		Validation 5 fold		Validation 10 fold	
	AUC	LL	UL	AUC	*p*-value	AUC	*p*-value	AUC	*p*-value
Type	0.825	0.777	0.872	0.818	0.795	0.819	0.827	0.812	0.590
Severity	0.949	0.924	0.974	0.950	0.924	0.947	0.906	0.946	0.828
During hospitalization	0.756	0.711	0.800	0.747	0.715	0.754	0.928	0.754	0.945
>4 drugs	0.739	0.685	0.793	0.732	0.804	0.728	0.677	0.721	0.512
>10 drugs	0.638	0.582	0.694	0.638	0.980	0.601	0.194	0.612	0.358

AUC, area under the curve; LL, lower limit of 95% confidence interval; *p*-value, statistical significance; UL, upper limit of 95% confidence interval; 4-fold, 4-fold cross validation; 5-fold, 5-fold cross validation; 10-fold, 10-fold cross validation.

## Discussion

### Incidence and Length of Stay

Very limited data exit on the incidence of DILI in the older population. The annual incidence of DILI has been estimated at between 19.1 and 2.7 cases per 100,000 adults (Sgro et al., 2002; Bjornsson et al., 2013; Vega et al., 2017). The incidence of DILI in patients over 65 years of age belonging our hospitals was 37.9 (95 CI 26.9–51.0) cases per 100,000 inhabitants. Older age appeared to be a risk factor for DILI in a 2-years Icelandic study, because the age-standardised incidence increased from 9 per 100,000 people in the group aged 15–29 years to 41 per 100,000 people in the group aged 80 years and older (Bjornsson et al., 2013). A higher proportion of older people were also found when analysing reports of DILI to WHO VigiBase database system, 62% of liver injury events reported were in adults aged 18–64 years, and 32% were in patients aged 65 years and older (Hunt et al., 2014).

As expected, in-hospital DILI were the most frequent (69%) in our study. A study using a Swiss database of hospitalised patients found that 1 in 100 patients developed DILI during hospitalisation (Meier et al., 2005). A recent single-centre, 1-year, prospective Colombian study reported that among hospitalised patients with elevated liver tests 6% had DILI (Cano-Paniagua et al., 2019). Hospitalised patients are regularly monitored for symptoms and by laboratory tests, in contrast, those patients with DILI ultimately resulting in hospitalisation could present abnormal liver function for weeks, which can go unidentified until the patients are admitted to a hospital. In accordance, a greater severity and worse outcome were observed in DILI resulting in hospitalisation in our study.

When comparing DILI by hospital department, internal medicine has the largest number (probably because it focused on the differential diagnosis), followed by traumatology, pneumology and haematology, in which DILI predominantly presented during hospitalisation, possibly due to the frequency of hepatotoxic drugs prescribed by these departments. The DILI causing hospitalisation were more frequently found in the gastroenterology department, as reported by De Valle (De Valle et al., 2006), which is likely due to the department’s specialisation in liver disease and the fact that their patients are often directly admitted from the emergency department. Unfortunately, the studies that included hospitalised patients, did not analyse the mean hospital stay or the prolongation of hospitalisations for DILI (Sgro et al., 2002; Meier et al., 2005).

### Characteristics of Patients With Drug-Induced Liver Injury

Our patients’ mean age was higher than that of other studies (Onji et al., 2009; Danjuma et al., 2020; Lucena et al., 2020), an important factor considering the age-related changes in body composition and hence in pharmacokinetics, which could be associated with increased risk and incidence. The elderly experience increased lipid-soluble drug distribution increased water-soluble drugs concentrations, and increased volume of drug distribution due to a decrease in serum albumin levels (Larrey, 2002; Lucena et al., 2020). In this sense, this study found that low albumin levels were associated with older age, greater severity and death from DILI. The male sex experienced greater severity and poorer outcomes and was associated with more smoking and alcohol consumption in this group. The age and alcohol consumption are well established risk factors for DILI (Danan and Benichou, 1993; Danan and Teschke, 2016; Danan and Teschke, 2018; Danan and Teschke, 2019). The female sex was also more frequent in the group over 75 years of age and was associated with a history of hypertension, dyslipidaemia and polypharmacy. Common chronic non-transmissible diseases that lead to polypharmacy in older people are the reason for the increase in the rate of adverse drug reactions in this population (Kowal et al., 2016; Ghabril et al., 2019). In a population-based case-control study using the UK General Practice Research Database, the risk of developing DILI was increased by a factor of six when a combination of two or more hepatotoxic drugs are present (de Abajo et al., 2004). In this study, pre-existing liver disease was associated to a greater severity of DILI. These results are in concordance with The DILIN Prospective Study in which DILI appeared to be more severe in patients (10%) with pre-existing liver disease (Chalasani et al., 2015).

### Characteristics of Drug-Induced Liver Injury Cases

As with our study, previous studies, have identified that the predominant pattern in DILI is hepatocellular (53.29%) (Friis and Andreasen, 1992; Larrey, 2002; Sgro et al., 2002; Meier et al., 2005; De Valle et al., 2006; Xu et al., 2012; Yeboah-Korang et al., 2020). Other studies, have observed varied results for the pattern (Bagheri et al., 2000; Ibanez et al., 2002; Andrade et al., 2005; Onji et al., 2009; Bjornsson et al., 2013), with a predominance of the cholestatic pattern, which was also associated with older age, which is probably related to a delay in the diagnosis, given that the symptoms are less manifest in the elderly (Onji et al., 2009), a situation that is avoidable with proactive pharmacovigilance. Regarding severity, (Aithal et al., 2011), it was observed that most cases had mild severity (65.07%), in accordance with previous studies (Meier et al., 2005; Lucena et al., 2009; Bjornsson et al., 2013; Douros et al., 2015).

Previous studies have frequently associated advanced age with persistent/chronic abnormalities due to a decrease in tissue repair function as the body ages Fontana et al., 2015). However, our results are in line with those of the study by Bjornsson and Davidsdottir (Bjornsson and Davidsdottir, 2009), which conducted a long follow-up of hospitalised patients, most of whose liver tests, normalised during follow-up, remaining free of liver morbidity and presenting a similar chronification rate to our study (1.53% vs. 1.2%).

Regarding the low rate of recorded DILI and hypertransaminasemia in the clinical discharge reports, there are numerous studies (Levy et al., 1999; Bagheri et al., 2000; Meier et al., 2005; De Valle et al., 2006; Xu et al., 2012) demonstrating a high rate of underestimation in the ADRs by spontaneous reporting, closely related to our study’s findings (35.96 and 57.89%, respectively). In a French population-based study, the number of hepatic events was 16 times greater than the number of spontaneously reported to the French authorities (Sgro et al., 2002). It was observed that the recorded rate depended on the severity and characteristics of the DILI, with more frequent registration in severe cases and for cholestatic patterns. However, our study had physicians from the Department of Clinical Pharmacology, who discuss the majority of cases with the attending physician. This approach, lead to an increase in the recorded rate of DILI in the discharge clinical reports, thereby demonstrating that if a retrospective analysis of only discharge reports had been conducted, 60–70% of cases would be lost (Meier et al., 2005) and that when a patient returns to the hospital, the new treating physician would not have all the patient’s information.

### Culprit Drugs

The top 5 drugs implicated in causing DILI in our study were, in descending order of frequency, paracetamol, amoxicillin-clavulanate, atorvastatin, cephazolin and levofloxacin. Except for drugs not indicated for older adult patients, our results were similar to those of a large DILI database (Teschke, 2018). In this study, paracetamol was the culprit drug for 11% (50/458) of DILI cases, one case was fatal. Due to this, in 2018 a recommendation for a maximum dose of paracetamol for the elderly of 3 g per day (previously 4 g daily) was implemented, achieving a reduction of 80% paracetamol related DILI 1 year after (data not shown). Ageing-related changes in liver blood flow and mass can increase paracetamol exposure causing more frequently acute liver injury with paracetamol given at therapeutic doses (Lucena et al., 2020). Prospective national and international DILI Registries have been set-up in Spain, United States, Europe, Latin American, Japan, and China among other countries to collect the most frequently implicated agents (Council for International Organizations of Medical Sciences, 2019). Antimicrobials (mainly amoxicillin-clavulanate) are the most frequent agents involved in DILI as reported in the Spanish DILI Registry (Andrade et al., 2006). Nevertheless, the amount of drugs consumed increases with age, and there will therefore be a parallel increase in the incidence of DILI (Meier et al., 2005).

It was also possible to calculate the incidence of DILI by drug consumed during hospitalisation, which was higher for piperacillin-tazobactam (66.96/10,000 DDDs), meropenem (56.6/10,000 DDDs) and atorvastatin (37.05/10,000 DDDs). In the study by Kang et al. (Kang et al., 2020), the authors provided the incidence rate per 1,000 prescribed patients with respect to piperacillin-tazobactam and meropenem (3.2 and 2.6, respectively). However, the incidence rate provided by of Kang et al. was for the entire population, which differed from our study rates were given only for those older than 65 years old. The incidence of atorvastatin use in LiverTox (National Institute of Diabetes and Digestive and Kidney Diseases, 2012) was very similar to that in our study (1: 3,000–1: 5,000), despite its use for the general population.

Up to date three prospective population-based studies have been published. A study carried out in France over a 3-years period the most frequent implicated drugs were NSAIDs, anti-infectious, psychotropic and hypolipidemic agents (Sgro et al., 2002). In Iceland, a 2-year period study amoxicillin-clavulanate, diclofenac and azathioprine were the most frequent causative agents (Bjornsson et al., 2013). More recently, in US, a 3-years study antibiotics and herbal and dietary supplements were the most frequent causative drug groups (Vega et al., 2017). Other studies have explored the causative drug groups of in-hospital DILI cases, being antiinfectives for systemic use, anticonvulsants or antineoplastic the most frequent implicated drugs (Meier et al., 2005; Cano-Paniagua et al., 2019). Antimicrobials and cardiovascular drugs were most frequently implicated in hepatotoxicity in older population in the Spanish DILI Registry and US Drug-induced Liver Injury Network (DILIN) (Lucena et al., 2009; Chalasani et al., 2015). In this sense, antiinfectives for systemic use, drugs for the central nervous system, and cardiovascular system drugs were found to be the therapeutic groups most frequently associated with DILI cases, both DILI resulting in hospitalisation and DILI in-hospital.

### Relevant Factors of Logistic Regression Models

In relation to the relevant factors included in the logistic regression models in terms of pattern and severity, the findings published in the literature by the Hy’s law and phenotype standardisation (Temple, 2006; Aithal et al., 2011) were confirmed. In the scientific literature, the RUCAM (Danan and Teschke, 2018) considers age a risk factor; however, no relationship between age and DILI in the explanatory models was found, despite the fact that the calculated incidence was greater than the general incidence reported in previous studies (Sgro et al., 2002; Bjornsson et al., 2013; Yang et al., 2017). Ethnicity has been reported as a risk factor, but unfortunately, it was not included in the data collection. Other risk factors such as alcoholism, chronic hepatitis, diabetes, human immunodeficiency virus infection, malnutrition, pregnancy and tuberculosis have been related to DILI but as risk contributors when associated with certain medications (Zimmerman, 1986; Bruno et al., 2005; Núñez, 2006; Rosenberg et al., 2007; Snijdewind et al., 2012). In our study, renal function impairment and the use of anti-infective drugs were found to be associated with DILI in polypharmacy and in-hospital DILI, respectively. Reduction of renal clearance with age is the most relevant and predictable change in drug pharmacokinetic, reducing the threshold dose needed to initiate cellular damage, especially in association with comorbidities and polypharmacy (Lucena et al., 2020). Antimicrobial ranks as the first causative drug class in several large cohorts of patients with DILI, and antimicrobial usage is much higher in older adult patients (Lucena et al., 2009; Bjornsson et al., 2013; Chalasani et al., 2015).

### Strengths and Limitations

Compared with the spontaneous reporting system (in which recognising an ADR can be difficult, only 1–10% of ADRs are spontaneously reported), the prospective pharmacovigilance programmes help to improve the detection of ADRs, the diagnosis of the causal drug and the reporting of ADRs, with high quality information on the ADRs and early identification before they can cause serious damage (Evans et al., 1994). The availability of clear denominators allows in pharmacovigilance programmes to calculate the incidence rate of ADRs. This study was conducted on an underrepresented population (older adults) and detected a specific type of adverse drug reaction (i.e., DILI) with a prospective follow-up using PPLSH. One of the study’s limitations lies in the fact that the data collection in the Getafe University Hospital was conducted only in the geriatric department, and therefore not over 65 years of age admitted to other departments were included, as in La Paz University Hospital. Secondly, there were different follow-up periods between the two hospitals, although when comparing them there were no statistical differences. Another important limitation is that certain DILI might have been lost in the search for alternative causes, as well as DILI attributed to an identifiable alternative cause. Our definition of ALS to detect DILI was based on the CDER-PhRMA-AASLD Conference, 2000 (EMEA, 2010). However, these parameters have been updated in 2011 because they were not specific enough to adequately detect clinically relevant liver lesions, prevent the inappropriate withdrawal of medications, and decrease the unnecessary study of hepatotoxicity, which could explain a higher incidence in our study, considering that the previous DILI definition had a higher sensitivity than the 2011 update (Aithal et al., 2011). Similarly, the RUCAM employed to assess the causality of the drugs was initially defined in the scientific literature in 1993 (Tegeder et al., 1999), and has been updated in 2016 (Danan and Teschke, 2016; Danan and Teschke, 2019), with the specification of grams of alcohol consumption, the inclusion of Hepatitis E virus biomarkers, and the addition of a simplified element management to further reduce inter-observer variability (Danan and Teschke, 2018). However, the updated 2016 RUCAM was not employed; the prospective data collection, paired with the extensive follow-up, the availability of a dedicated clinical pharmacologist and the sensitivity of the treating physicians involved in the data collection conferred a high-quality causality classification method to our study.

## Conclusion

Through PPLSH we were able to follow-up a specific population in an ADR of interest (DILI in elderly patients). A higher incidence of DILI, mild severity, prolonged hospital stay, good outcome, and a hepatocellular pattern, with 72.7% of the DILI developing during hospitalisation were found. Also, the general incidence of DILI per hospitalisation, and the most frequent causal drugs (paracetamol, amoxicillin-clavulanate and atorvastatin) were reported. This type of proactive drug surveillance favours the creation of explanatory models of risk factors and helps to better monitor DILI in some of the most vulnerable and underrepresented populations. This surveillance not only increases our knowledge of DILI in this population but also improves their detection, the diagnosis of the culprit drugs and the notification of DILI.

## Data Availability Statement

The data that support the findings of this study are available contacting the corresponding authors.

## Author Contributions

The three corresponding authors (LP, JAC, and ER) participated in the design, data collection, statistical analysis and writing of this article. OL and LR-M participated in the design of the study and the article writing. DG-R participated in the writing and edition. JF participated in the design.

## Conflict of Interest

The authors declare that the research was conducted in the absence of any commercial or financial relationships that could be construed as a potential conflict of interest.
